# Roles of the Wnt Signaling Pathway in Head and Neck Squamous Cell Carcinoma

**DOI:** 10.3389/fmolb.2020.590912

**Published:** 2021-01-05

**Authors:** Jing Xie, Li Huang, You-Guang Lu, Da-Li Zheng

**Affiliations:** ^1^Fujian Key Laboratory of Oral Diseases, School and Hospital of Stomatology, Fujian Medical University, Fuzhou, China; ^2^Department of Preventive Dentistry, School and Hospital of Stomatology, Fujian Medical University, Fuzhou, China; ^3^Department of Dentistry, The First Affiliated Hospital of Fujian Medical University, Fuzhou, China

**Keywords:** Wnt signaling pathway, head and neck squamous cell carcinoma, canonical, non-canonical, epigenetic

## Abstract

Head and neck squamous cell carcinoma (HNSCC) is the most common type of head and neck tumor. It is a high incidence malignant tumor associated with a low survival rate and limited treatment options. Accumulating conclusions indicate that the Wnt signaling pathway plays a vital role in the pathobiological process of HNSCC. The canonical Wnt/β-catenin signaling pathway affects a variety of cellular progression, enabling tumor cells to maintain and further promote the immature stem-like phenotype, proliferate, prolong survival, and gain invasiveness. Genomic studies of head and neck tumors have shown that although β-catenin is not frequently mutated in HNSCC, its activity is not inhibited by mutations in upstream gene encoding β-catenin, NOTCH1, FAT1, and AJUBA. Genetic defects affect the components of the Wnt pathway in oral squamous cell carcinoma (OSCC) and the epigenetic mechanisms that regulate inhibitors of the Wnt pathway. This paper aims to summarize the groundbreaking discoveries and recent advances involving the Wnt signaling pathway and highlight the relevance of this pathway in head and neck squamous cell cancer, which will help provide new insights into improving the treatment of human HNSCC by interfering with the transcriptional signaling of Wnt.

## Introduction

Head and neck squamous cell carcinoma (HNSCC) is the sixth most common malignant tumor in the world (Alamoud and Kukuruzinska, 2018). HNSCC causes over 330,000 deaths worldwide, and more than 650,000 HNSCC cases are reported each year (Xi and Grandis, 2003). In the United States, the overall incidence of HNSCC is 11 per 100,000 people, and HNSCC is more common among black populations than white populations. It originates from the mucosa of various organs that have a squamous epithelial lining. These organs include the mouth, nasopharynx, and throat. Oral squamous cell carcinoma (OSCC) is the main type of HNSCC, which is characterized by poor prognosis and low survival rate. Local recurrence of the primary site and cervical lymph node metastasis are the main reasons for the failure of treatment in patients with OSCC. Therefore, elucidating the molecular mechanisms that regulate the occurrence and development of OSCC will help to understand the etiology of these diseases, allow the design of more effective strategies for the treatment of OSCC, and possibly improve treatment.

In 1982, Nusse found an oncogenic gene in mouse models of mammary cancer, named int1, and which has homology to the wingless gene of drosophila reported later by Sharma, and the two were collectively called Wnt (Nusse et al., 1991). The Wnt signaling pathways play important roles in embryonic development, tissue regeneration, cell proliferation, and cell differentiation and is abnormally activated in many types of cancers, such as colon cancer (Zheng and Yu, 2018; Flores-Hernández et al., 2020), liver cancer (Li et al., 2019), lung cancer (Ji et al., 2019), breast cancer (Ma et al., 2016), and childhood T-cell acute lymphoblastic leukemia (Ng et al., 2014). Previous studies have shown that dysfunction of the Wnt signaling pathway can promote the development of oral cancer (González-Moles et al., 2014) and that abnormalities in this pathway affect the prognosis of patients with HNSCC. More and more research highlights the importance of the Wnt signaling pathway for the prognosis of HNSCC patients and suggests the possibility of actively developing new gene therapy methods that target this pathway in HNSCC. Thus, this review summarizes recent research findings regarding the Wnt signaling pathway in HNSCC to improve our understanding of the mechanisms underlying the roles of this important signaling pathway in cancer cell activity.

## Wnt Signaling Pathway

With the advancement of research, people are learning more and more about the Wnt signaling pathway. So far, 19 members of the Wnt family have been found in the human genome, including Wnt1, Wnt2, Wnt2b, Wnt3, Wnt3a, Wnt4, Wnt5a, Wnt5b, Wnt6, Wnt7a, Wnt7b, Wnt8a, Wnt8b, Wnt10a, Wnt10b, Wnt11, Wnt14, Wnt15, and Wnt16. These secreted glycoproteins usually contain 350–400 amino acids. In order to trigger the cellular response and activate intracellular signal transduction, the extracellular Wnt ligands combine with the 10 Frizzled (Fzd 1-10) receptors and several coreceptors, such as Lrp-5/6, Ryk, or Ror2 (Logan and Nusse, 2004; Kestler and Kühl, 2008). Intracellular signal transduction cascades diversify into three main branches, the canonical Wnt/β-catenin signaling pathway, and the non-canonical Wnt signaling pathway, which mainly comprises the Wnt/Ca^2+^ and Wnt/PCP pathways (González-Moles et al., 2014).

## Canonical Wnt Signaling Pathway

The hallmark of the canonical Wnt signaling pathway is the accumulation and transport of β-catenin proteins associated with adhesion junctions into the nucleus (Dawson et al., 2013). In an experimental analysis of the axial development of *Xenopus laevis* and the segmental polarity and wing development of Drosophila, researchers first clarified the role of this canonical pathway in embryonic development (Ng et al., 2019). glycogen synthase kinase 3 (GSK3)β is a central participant in the canonical Wnt pathway. The activity of the Wnt/β-catenin signaling pathway depends on the amount and cellular location of β-catenin (Lustig and Behrens, 2003). Wnt ligands interact with the Fzd receptors. When the Fzd receptors are unoccupied, cytoplasmic β-catenin is degraded by its destruction complex, which includes Axin, APC protein, GSK3, casein kinase 1α (CK1α), and β-catenin (Tejeda-Muñoz and Robles-Flores, 2015). Once the complex is formed, β-catenin begins to phosphorylate sequentially. The first phosphorylation is at Ser45 by CK1α, and subsequently at Thr41, Ser37, and Ser33 by GSK3β. Phosphorylated β-catenin is released from the complex allowing for its ubiquitination at the N-terminal end of the protein and subsequent degradation by E3. Axin and APC can also be phosphorylated by GSK3β and CK1α, resulting in the enhancement of β-catenin phosphorylation (Hagen and Vidal-Puig, 2002). This continuous degradation prevents the accumulation and translocation of β-catenin to the nucleus (MacDonald et al., 2009). When the Wnt/β-catenin signaling is activated, Wnt ligand binds to Fzd receptors and its co-receptor, low-density lipoprotein receptor-related protein 5/6 (Lrp5/6) (Gordon and Nusse, 2006). This complex leads to the recruitment of the scaffold protein (Disheveled, Dvl) to the receptors which are then phosphorylated. Subsequently, Axin, GSK3β, and CK1 migrate from the cytoplasm to the plasma membrane, which contributes to the inactivation of the destruction complex, resulting in β-catenin stabilization through dephosphorylation. Stable β-catenin translocates into the nucleus and interacts with T-cell factor (TCF) transcription factors to induce the expression of Wnt target genes such as c-Myc, cyclin D1, Axin-2, Lgr5, ITF-2, PPAR-δ, and matrix metalloproteinase 1 and 7 (MMP-1, MMP-7) (Wu and Pan, 2010; Velázquez et al., 2017). A variety of Wnt/β-catenin target genes have been identified, including cell proliferation regulation genes, development control genes, and genes related to tumor progression. Wnt1 class ligands (Wnt2, Wnt3, Wnt3a, and Wnt8a) play main roles through the canonical Wnt/β-catenin signaling pathway.

## Non-Canonical Wnt Signaling Pathway

Non-canonical Wnt signaling is mediated through Fzds but Lrp5/6 is not involved and consists of two main branches (Valenta et al., 2012): the PCP pathway and the Wnt/Ca^2+^ pathway. Non-canonical Wnt signaling is initiated by Wnt5a type ligands (Wnt4, Wnt5a, Wnt5b, Wnt6, Wnt7a, and Wnt11). These Wnt ligands bind to Fzd receptors. In addition, receptor tyrosine kinase-like orphan receptor 2 (Ror2), and receptor tyrosine kinase (Ryk) have been suggested as non-canonical signaling co-receptors, which are required for downstream activation. These signal transductions jointly activate the calcium-dependent signaling cascade by activating Dvl (Rao and Kühl, 2010). In the Wnt/Ca^2+^ pathway, Wnt ligands bind to receptor complex, leading to the activation of phospholipase C (PLC). This results in inositol 1,4,5-triphosphate-3 (IP3) production and subsequent Ca^2+^ release (Anastas and Moon, 2013). Calcium release and intracellular accumulation activate several calcium-sensitive proteins, including protein kinase C (PKC) and calcium/calmodulin-dependent kinase II (CaMKII) (González-Moles et al., 2014). Calcineurin activates nuclear factor of activated T cells (NFAT) and subsequent NFAT-mediated gene expression (Saneyoshi et al., 2002). Some evidence had been found that parts of the non-canonical Wnt signaling proteins influence the canonical Wnt/β-catenin pathway (van Tienen et al., 2009; Fan et al., 2017). However, the specific mechanism is not yet clear, and more research is needed.

**FIGURE 1:**
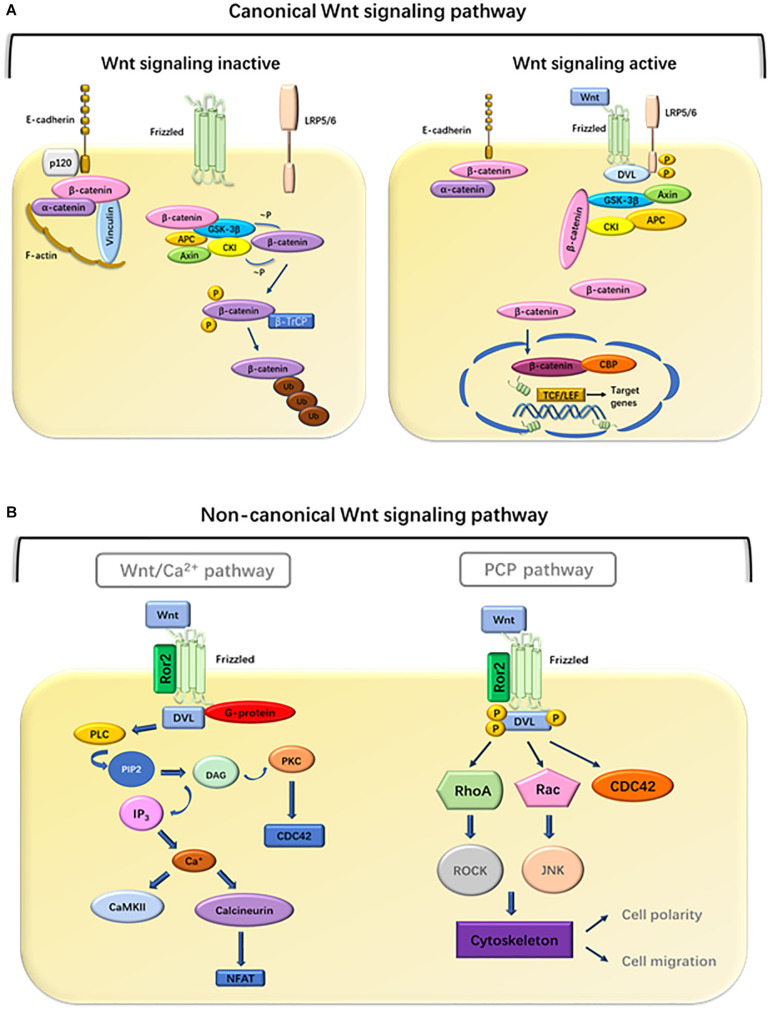
Overview of the Wnt pathway. **(A)** Canonical pathway. Binding of Wnt to frizzled receptors activates disheveled (DVL), which disrupts the stability of the destruction complex, composed of Axin, APC, GSK3-β, CK1, and β-catenin. Subsequently, phosphorylation and degradation of β-catenin are inhibited, which allows the association of β-catenin with TCF transcription factors. In the absence of Wnt ligands, the complexes promote phosphorylation of β-catenin. Phosphorylated β-catenin becomes multiubiquitinated (Ub) and subsequently degraded in proteasomes (Foulquier et al., 2018). **(B)** Non-canonical pathway. In the Wnt/Ca^2+^ pathway, Wnt ligands bind to a complex consisting of Fzd, DVL, and G-proteins, leading to the activation of PLC, which cleaves phosphatidylinositol 4,5 biphosphate (PIP2) into diacylglycerol (DAG) and IP3. DAG activates PKC whereas IP3 promotes the release of intracellular Ca^2+^, which in turn activates CamKII and calcineurin (Russell and Monga, 2018). Calcineurin activates NFAT to regulate cell migration and cell proliferation. In the PCP pathway, Wnt ligands bind to a complex consisting of Fzd, Ror2, and DVL, which mediates the activation of RhoA and ROCK, or activation of Rac and JNK signaling, to regulate cell polarity and migration.

PCP was first demonstrated in insects because their cuticular surface has a rich morphology (Adler, 2012). The Wnt/PCP pathway mediates the event of collective migration, but abnormal activation leads to tumor migration ability. In the Wnt/PCP pathway, the binding of Wnt to Fzd and a co-receptor causes recruitment of Dvl to Fzd and its association with disheveled-associated activator of morphogenesis 1 (DAMM1). DAMM1 activates small G protein Rho, through guanine exchange factor and then activates Rho-associated protein kinase to reorganize the cytoskeleton and change cell polarity and migration (Peng et al., 2011). It is characteristic of the plane polarity signal that Rho-associated kinases can mediate cytoskeleton rearrangement. Alternatively, the PCP pathway can also be mediated by the triggering of RAC to initiate the c-Jun amino terminal kinase (JNK) signaling cascade (Javed et al., 2019). The activation of Dvl-mediated Wnt signal induces the activation of heterotrimeric G protein and promotes the transport of intracellular Ca^2+^ to the extracellular environment (De, 2011). This transport activates JNK and Nemo-like kinase (NLK) which can phosphorylate TCF transcription factors and antagonize the canonical Wnt signaling pathway (Humphries and Mlodzik, 2018). Taken together, these observations indicate that the Wnt/Ca^2+^ pathway is a key regulator of canonical signaling pathways and planar cell polarity pathways. On the other hand, non-canonical signaling pathways phosphorylate TCF through NLK, thereby mediating the activation of canonical Wnt signaling (Figure 1).

## Aberrant Wnt Signaling Pathway in HNSCC

With the discovery that a number of Wnt genes are associated with the development of various human cancers, aberrant activation of Wnt signaling pathway became evident. To date, different roles of Wnt in HNSCC have been confirmed. Leethanakul et al. used microarray technology to reveal the role of Wnt in HNSCC for the first time. They found that homologs of both Fzd and Dvl were increased compared with normal tissue samples. This suggests that Wnt mediates invasiveness in the development of HNSCC (Leethanakul et al., 2000). Currently, several other studies have shown that abnormal activation of the Wnt signaling pathway facilitates tumor transformation in head and neck tissues (Iwai et al., 2005). For example, Wnt1-induced signaling pathway protein 1 (WISP-1) is involved in the progression of OSCC, and high expression of WISP-1 is significantly associated with treatment failure (Zhang C. et al., 2019). Wnt7b, an agonist of the canonical Wnt pathway, shows significantly increased expression in samples from patients with OSCC compared with matched samples of adjacent non-tumorous tissues (Shiah et al., 2016), and the Wnt/β-catenin signaling pathway prevents shedding-mediated apoptosis (anoikis) in SCC1 cells and promotes the growth of HNSCC-xenograft tumors *in vivo* (Farooqi et al., 2017). The Wnt/β-catenin signaling pathway may regulate the epithelial–mesenchymal transition in laryngeal squamous cell carcinoma, thereby regulating tumor development (Psyrri et al., 2014). In OSCC, the non-canonical Wnt/Ca^2+^/PKC pathway is activated by Wnt5a, which promotes migration and invasion (Prgomet et al., 2015). Wnt5b has been found to be significantly increased in the highly metastatic cell line of OSCC cells. Wnt5b gene silencing can significantly inhibit the formation of filopodia-like protrusive structures and migration, whereas stimulation with Wnt5b can significantly increase the formation of filopodia-like protrusions in SAS-LM8 cells (Takeshita et al., 2014). The roles of more Wnt ligands in HNSCC are listed in Table 1. Thus, both canonical Wnt pathways and non-canonical Wnt pathways play great roles in HNSCC. Although Wnt1 type or Wnt5a type ligands activate canonical or non-canonical Wnt pathways, respectively, there is more research that suggests that the results of different Wnt ligands depend on specific combinations of Wnt receptors and coreceptors (Wang et al., 2013; Sakisaka et al., 2015). Besides the canonical Fzd and Lrp receptor, Ror and Ryk are also important alternative receptors for Wnt transduction.

**TABLE 1:** The roles of different Wnt ligands in HNSCC.

Wnt ligands	Type of Wnt signaling	HNSCC Cell lines	Type of HNSCC	Function	References
Wnt1	Canonical	SCC1483, SNU1076	Oral squamous cell carcinoma	Promote invasion, inhibit apoptosis	Rhee et al., 2002; Zhang C. et al., 2019
Wnt3	Canonical	–	Oral leukoplakia	Cause dysplasia	Ishida et al., 2007
Wnt3a	Canonical	–	Laryngeal squamous cell carcinoma	Worse histological grade, advanced clinical stage, and higher cervical lymph node metastatic potential	Zhang D. et al., 2019
Wnt4	Non-canonical	WRO, CAL62, FB2, and BCPAP	Thyroid carcinoma	Reduce migration	Filippone et al., 2014
Wnt5a	Non-canonical	SCC9	Oral squamous cell carcinoma	Enhance migration and invasion	Prgomet et al., 2017
Wnt5a	Non-canonical	HTH-74, C-643	Thyroid carcinoma	Decrease proliferation, migration, invasiveness, and clonogenicity	Kremenevskaja et al., 2005
Wnt5a	Non-canonical	CNE-2, 5-8F	Nasopharyngeal carcinoma	Lead to tumorigenesis and metastasis	Zhu et al., 2014; Qin et al., 2015
Wnt5a	Non-canonical	–	Laryngeal squamous cell carcinoma	High tumor stage and lymph node metastasis	Prgomet et al., 2017
Wnt5b	Non-canonical	SAS-LM8	Oral squamous cell carcinoma	Enhance migration and invasion	Zhang et al., 2017
Wnt7a	Canonical	HSC3, CAL27	Oral squamous cell carcinoma	Promote migration	Sakamoto et al., 2017
Wnt7b	Canonical	DOK, FaDu	Oral squamous cell carcinoma	Promote proliferation and invasion	Xie et al., 2020
Wnt10b	Canonical	SNU1076	Head and neck squamous cell carcinoma	Promote growth and survival, inhibit apoptosis	Shiah et al., 2014
Wnt11	Non-canonical	–	Oral squamous cell carcinoma	Suppress tumor	Andrade Filho et al., 2011

Head and neck squamous cell carcinoma can be divided into human papillomavirus (HPV)-positive and HPV-negative tumors, each of which has its unique clinical, pathological, and epidemiological significance (Cancer Genome Atlas Network, 2015). Increasing evidence shows that Wnt/β- catenin signaling has an impact on the pathobiology of HPV- and HPV + HNSCC. HPV viral oncoprotein E6/E7 has been used to alter the prognosis of HPV-HNSCC patients (Liu et al., 2017). In oropharyngeal squamous cell carcinoma, β-catenin is driven to nuclear translation through E6 oncoprotein by activating epidermal growth factor receptors (EGFR). Some researchers have used small interfering RNAs to suppress E6 expression and erlotinib to downregulate EGFR activity and thereby eliminate the nuclear localization of β-catenin and the phosphorylation of EGFR while reducing the invasion characteristics of HPV + HNSCC cell lines *in vitro* (Nwanze et al., 2015). According to reports, E6/E7 may also suppress E3 ubiquitin ligase protein to induce nuclear translocation of β-catenin. The regulatory effect of E6/E7 on HPV + HNSCC requires further study. Recently, it was found that some microRNAs have potential roles in the attenuation of HPV+/HPV- HNSCC, although the effects are weak (Nwanze et al., 2015). More research is needed to deepen the understanding of the Wnt/β-catenin signaling pathway in HPV + HNSCC (Kobayashi et al., 2018). Due to limited tumor specimens and relevant clinical data, research on HPV + HNSCC lags behind than on HPV-HNSCC (Cancer Genome Atlas Network, 2015; Beck and Golemis, 2016).

## Genetic and Epigenetic Changes of Wnt Signaling in HNSCC

Components of the Wnt signaling pathway, such as Wnt ligand proteins, Wnt antagonists, membrane receptors, and intracellular conduction medium, are often disrupted by genetic or epigenetic inheritance in human tumors (Polakis, 2012). It is reported that the activation of the Wnt1 and Wnt pathways occurs due to epigenetic changes in secreted frizzled-related protein (SFRP), Wnt inhibitory factor (WIF), and the Wnt signaling pathway inhibitor Dickkopf 3 (DKK3). Previous data demonstrated that DKK-3 protein is mainly expressed in HNSCC (Katase et al., 2013), and its expression is associated to the high metastasis rate and poor prognosis of OSCC (Katase et al., 2012). Therefore, epigenetic changes of DKK3 may be closely related to the occurrence and development of HNSCC (Katase et al., 2020). Epigenetic alterations of SFRP, WIF-1, and DKK-3 genes can active Wnt pathways, resulting in delocalization of catenin in HNSCC (Pannone et al., 2010). It was recently reported that overexpression of β-catenin is significantly associated with increased transcriptional activity in HNSCC (Kartha et al., 2018). The destructive complex strictly controls the level of β-catenin in the cytoplasm. Previous studies have suggested that mutations in APC, Axin, and β-catenin are widespread in colon cancer (Hernández-Maqueda et al., 2013; Yu et al., 2018), esophageal cancer, and gastric cancer. The Axin1 mutation was first identified in hepatocellular carcinoma (Satoh et al., 2000). In a small, diverse group of colon cancer cases, activation of point mutations in β-catenin removed the regulated N-terminal Ser/Thr residue. Similar β-catenin mutations have also been reported in melanoma and other tumors (Morin et al., 1997; Rubinfeld et al., 1997). Mutations in these genes stabilize β-catenin, allowing it to accumulate in the nucleus, and subsequently activate the Wnt signaling pathway. However, mutants of APC, Axin, or β-catenin still ultimately depend on exogenous Wnts (Lammi et al., 2004). According to HNSCC studies, there are few gene mutations relevant to Wnt pathways in HNSCC, which indicates that abnormal β-catenin accumulation in oral cancer is not associated with mutations in these genes. Although Wnt/β-catenin mutations are not common in HNSCC, other signal pathways, such as FAT1 and AJUBA, can crosstalk with Wnt/β-catenin, resulting in changes in the activity of Wnt signaling pathway (Cancer Genome Atlas Network, 2015; Beck and Golemis, 2016). Mutations in these signaling cascades are almost entirely related to HPV-negative tumors and to the absence of epithelial differentiation programs. Another possible mechanism for the degradation and inactivation of β-catenin involves EGFR signaling (Lee et al., 2010). In OSCC, EGFR stabilizes β-catenin and enhances nuclear accumulation of β-catenin through phosphorylation, possibly via two molecular mechanisms: (1) binding directly and then β-catenin is phosphorylated and (2) phosphorylation through GSK-3β to regulate the activity of the destruction complex (Billin et al., 2000; Hu and Li, 2010).

DNA methylation and histone modification also play important parts in the occurrence of HNSCC. Epigenetic regulation may contribute to the silencing of Wnt related genes. Because there is no changes of methylation levels in the CpG island of APC, Axin, and β-catenin genes in OSCC (Shiah et al., 2016), downregulation of Wnt signaling in OSCC and HNSCC is usually due to methylation of different Wnt pathway inhibitors, such as SFRP-2, WIF-1, DKK-1 (Katase et al., 2010), Dachshund family transcription factor 1 (DACH1), and RUNT-related transcription factor 3 (RUNX3). Microarray-based genome-wide epigenetic analyses of human cancer have shown that inhibitors of Wnt signaling pathway are common sites for promoter methylation silencing. However, these Wnt pathway inhibitors may have different levels of methylation in OSCC and HNSCC cells, and may be significantly related to tumor recurrence or disease-free survival. For example, in OSCC cell, the WIF-1 and SFRP2 genes are frequently methylated, whereas the DACH1 and Dkk1 genes are less frequently methylated (Farooqi et al., 2017). In the same way, the WIF-1 gene is often methylated in primary oropharyngeal cancer tissue and associated with poorer survival (Paluszczak et al., 2015). In addition, methylation of the E-cadherin promoter is the main reason for the loss of membrane β-catenin expression, which leads to the release of β-catenin from the E-cadherin/β-catenin complex into the cytoplasm (Wong et al., 2018). By performing chromatin immunoprecipitation promoter array and gene expression analyses in hepatocellular carcinoma, Cheng et al. (2011) found that enhancer of zeste homolog 2 (EZH2) occupancy of the promoter decreased the expression of several Wnt antagonists including Axin2, NKD1, PPP2R2B, DKK1, and SFRP5. EZH2 is the core components of polycomb repressor complex 2 (PRC2) and has methyltransferase activity. It can catalyze histone 3 lysine 27 trimethylation (H3K27me) and eliminate PRC2-mediated gene suppression. Thus, overexpression of EZH2 promotes the neoplastic transformation of epithelial cells. These findings show that inhibiting the activity of Wnt antagonists through DNA methylation and histone modification enables to the constitutive activation of Wnt/β-catenin signaling. Moreover, testing body fluids to detect DNA methylation is feasible and minimally invasive. Therefore, the Wnt antagonist gene such as SFRP-2, WIF-1, and DKK-1 secreted in plasma can be used as a biomarker for diagnosis and prognosis (Shiah et al., 2016).

## Wnt Signaling Pathway in Cancer Stem Cells of HNSCC

Stem cells (SCs) have the ability of self-renewal and differentiation. The maintenance and repair of tissue homeostasis depends on the activity of tissue-specific SCs. Cancer SCs (CSCs) are a subset of cells that are resistant to chemotherapy and radiotherapy and often promote relapse by stopping or evading clinical treatment (Mannelli and Gallo, 2012). Like other cancer tissues, HNSCC tissue contains small cell subsets with stem-like characteristics (CSCs), which can bring about tumors with hierarchical structure.

According to reports, aberrant Wnt signaling has a promoting effect on different forms of cancer (such as colon cancer, liver cancer, and lung cancer), and plays a key role in guarding CSCs (Vermeulen et al., 2010). Le et al. co-cultured HNSCC tumor spheres and cancer-related fibroblast (CAF) cell line in 3D environment to simulate the interaction *in vivo* and found that Wnt3a activated Wnt signals in cancer cells and CAF. The activation of Wnt increases the characteristics of CSC, such as sphere formation and invasiveness (Lamb et al., 2013; Le et al., 2019). Non-canonical Wnt signals in CSCs are activated by Wnt5a, Wnt11, or other non-canonical Wnt ligands. It is known that non-canonical Wnt signals promote the survival and drug resistance of CSCs through activation of PI3K-AKT signal and YAP/TAZ-mediated transcription. But there are few studies on the role of non-canonical Wnt signaling pathway in the CSC of HNSCC, most of the findings focus on the Wnt/β-catenin signaling pathway. Recent advances suggest that Wnt/β-catenin signaling is involved in the differentiation and development of CSCs in HNSCC. One proposed mechanism is that Wnt/β-catenin may play a specific role in asymmetric cell division, which allows Dvl, Fzd, Axin, and APC to divide asymmetrically in the cytoplasm, producing a progenitor cell and a cell destined to differentiate (Lien and Fuchs, 2014). The analysis of CSC proliferation stimulated by canonical Wnt signal pathway inhibitors has become the latest experimental method to study the role of this signal pathway in CSC self-renewal. In nasopharyngeal carcinoma, CSC isolated from HNE1 cell line treated with Wnt-C59, an inhibitor of Wnt, can reduce the proliferation of CSC (Cheng et al., 2015). In addition, several other studies have shown that numerous canonical Wnt signal pathway inhibitors, including SFRP4, all-trans retinoic acid (Atra), and active natural compounds and honokiol, can reduce the expression of β-catenin and ultimately inhibit the proliferation of CSC in HNSCC (Lim et al., 2012; Yao et al., 2017). The Wnt/β-catenin signaling pathway also plays an important role in regulating differentiation of SC during early embryonic development (Vlad et al., 2008) and cancer including HNSCC. It is reported that CSC isolated from M3a2 and M4e (HNSCC cell lines) are highly activated. The CSCs injected into nude mice differentiate into tumor cells, resulting in five times larger tumor growth than non-CSC after 8 weeks (Lee et al., 2014).

A study showed that the expression of CD44 + was essential for maintaining tumor heterogeneity in HNSCC (Prince et al., 2007). The CSCs with high CD44+ were shown to be characterized by high aldehyde dehydrogenase activity (ALDH) and by expression of c-Met and SOX2. According to reports, CD44+/ALDH (high) cells have stronger oncogenicity and self-renewal ability than CD44 + ALDH (low) cells. ALDH is thought to cause treatment resistance and tumor prevalence by regulating the expression of phosphoinositide 3-kinase (PI3K) and SOX2 signaling pathway (Bertrand et al., 2014). The mesenchymal–epithelial transition factor c-Met has been reported to interact with the Wnt/β-catenin pathway in HNSCC (Arnold et al., 2017). The roles of c-Met and Wnt/β-catenin have been widely studied in colon cancer cells, in which their activities determine the fate of cells in CSC. However, the activation of c-Met inhibitor in the presence of β-catenin has been found to result in the elimination of CSCs in HNSCCs (Arnold et al., 2017). It has been reported that FZD8, a modulator of the Wnt/β-catenin pathway, increases the expression of CSCs in HNSCCs by activating the (extracellular regulated MAP kinase) ERK/c-fos signaling axis (Bordonaro et al., 2016; Chen and Wang, 2019).

Due to the presence of drug-resistance CSCs, disease recurrence is the main marker of HNSCC. A large body of evidence suggests that Wnt confers chemotherapeutic resistance by upregulating CSC activity in HNSCC. The use of the Fzd/Wnt antagonist SFRP4 was found to increase the drug sensitivity of HNSCC by 25%. SFRP4 was shown to compete directly with Wnt, significantly enhancing cisplatin-induced apoptosis and reducing the activity of tumor cells (Warrier et al., 2014). Furthermore, the use of antagonists had no effect on non-tumorigenic mouse embryonic fibroblasts, suggesting that Wnt signaling plays an important role in the development and differentiation of CSCs related to HNSCC. However, the potential mechanism underlying the upregulation of chemical resistance in CSCs remains unclear, as does the mechanism by which Wnt mediates the activation of CSCs. Studies have identified five types of ABC transporters, ABCC1 to ABCC5, as main mediators in the canonical hyperactivation of the Wnt pathway in spheroid cells of HNSCC. The ability of spheroid cells to exhibit CSC-induced chemotherapy resistance was eliminated after knocking out the genes for β-catenin synthesis. However, this knock out resulted in the loss of SC tags necessary for self-renewal (Song et al., 2010; Yao et al., 2013). Although research on Wnt signal modulators has made great progress, few drugs have been imported for clinical use. Since CSCs have the same characteristics (self-renewal, differentiation) as normal SCs, they present an obstacle to the development of suitable pharmaceutical formulations for HNSCC.

## Wnt Signaling as a Therapeutic Target for HNSCC

Wnt signaling plays an important role in tumorigenesis and acts as a regulator of CSCs renewal in the process of cell homeostasis; thus, it is an attractive therapeutic target. To date, several approaches have been developed, and a few have moved on to clinical trials. One of them is to block the activity of Wnt with specific inhibitor. PORCN, also known as porcupine, is an enzyme which can limit the activation of Wnt signals in serine residues and promote the palmitoylation of Wnt. Using small inhibitors of PORCN, such as IWP, C59, and LGK974 caused rapid decreases in the expression of Wnt signaling (Proffitt et al., 2013). *In vitro*, C59 inhibited the activity of PORCN, and then inhibited the Wnt palmitoylation, Wnt interaction with carrier protein Wntless/WLS, Wnt secretion, and Wnt activation of β-catenin reporter protein. The chick chorioallantoic membrane (CAM) experiment proved that LGK974 can inhibit the growth and metastasis of HNSCC (Rudy et al., 2016). Studies have also shown that PORCN directly prevents the excessive production of Wnt, thus inhibiting the interaction between Wnt and Fzd protein. At present, the inhibition of PORCN on Wnt is being verified *in vivo* and *in vitro*. Additionally, inhibitors of tankyrase stabilize axin and antagonize Wnt signaling including XAV939, IWR, G007-LK, and G244-LM, though they have not yet entered clinical trials (Huang et al., 2009; Lau et al., 2013; Kulak et al., 2015). Moreover, ICG-001, a small molecule that inhibits the transcription of CREB binding proteins, downregulates β-catenin/T cell factor signaling by specifically binding to cyclic AMP response element-binding protein (Emami et al., 2004; Bordonaro and Lazarova, 2015). ICG-001 is currently in phase I clinical trials in patients with HNSCC. Furthermore, OMP-18R5 is a human monoclonal antibody against the Fzd receptor and is currently in phase I clinical trials. Wnt ligands and their compound receptors are also being evaluated in clinical trials (Kawakita et al., 2014). Examples include Omp-54F28, a chimera of human IgG1 and Fzd8, which is related to the growth of pancreatic cancer cells. Currently, most clinical trials use small RNAs as biomarkers for cancer detection, diagnosis, and prognostic evolution (Hayes et al., 2014). To date, no clinical trial has used miRNAs to predict prognosis and the clinical effect in HNSCC patients. A more comprehensive understanding of the involvement of the Wnt pathway in HNSCC is necessary to develop effective therapeutics for oral cancer.

## Conclusion

As outlined above, aberrant activation of the Wnt signaling pathway may impact on HNSCC. In addition to gene mutations in the Wnt component, abnormal changes downstream of EGFR are involved in regulating the Wnt/β-catenin pathway, which can reshape the histone/chromatin structure of the target gene. Because the epigenetic alterations of Wnt antagonists are the cause of Wnt signal activation, it may become a potential biomarker for predicting OSCC recurrence in plasma. Appropriate methods are required to deal with CSC generated by aberrant Wnt signaling. Wnt signaling is one of the regulators of CSC generation involving HNSCC. Because of the complexity of non-canonical signal pathway, most of the research on Wnt in HNSCC is focused on canonical WNT signal pathway, but there are few related studies on non-canonical signal pathway. More attention needs to be paid to non-canonical signaling pathways in the future. The evaluation of various aspects of signal transduction can expand our understanding of both this key pathway and the crosstalk between signaling pathways in cells. Such advancement will enable the development of a broad range of therapeutic interventions to eradicate and respond to HNSCC recurrence.

## Author Contributions

JX and LH contributed equally in conceiving the review focus, conducting the literature review, summarizing the manuscript, writing the first draft, and finalizing the manuscript. D-LZ and Y-GL designed and directed the review. JX, LH, D-LZ, Y-GL revised and made corrections to the manuscript. All authors have read and agreed to the final version of the manuscript.

## Conflict of Interest

The authors declare that the research was conducted in the absence of any commercial or financial relationships that could be construed as a potential conflict of interest.

## References

[B1] Adler P. N. (2012). The frizzled/stan pathway and planar cell polarity in the *Drosophila* wing. *Curr. Top. Dev. Biol.* 101 1–31. 10.1016/B978-0-12-394592-1.00001-6 23140623 PMC3575171

[B2] Alamoud K. A. Kukuruzinska M. A. (2018). Emerging insights into Wnt/β-catenin signaling in head and neck cancer. *J. Dent. Res.* 97 665–673. 10.1177/0022034518771923 29771197 PMC11066518

[B3] Anastas J. N. Moon R. T. (2013). WNT signalling pathways as therapeutic targets in cancer. *Nat. Rev. Cancer* 13 11–26. 10.1038/nrc3419 23258168

[B4] Andrade Filho P. A. Letra A. Cramer A. Prasad J. L. Garlet G. P. Vieira A. R. (2011). Insights from studies with oral cleft genes suggest associations between WNT-pathway genes and risk of oral cancer. *J. Dent. Res.* 90 740–746. 10.1177/0022034511401622 21393552 PMC3092817

[B5] Arnold L. Enders J. Thomas S. M. (2017). Activated HGF-c-met axis in head and neck cancer. *Cancers* 9:169. 10.3390/cancers9120169 29231907 PMC5742817

[B6] Beck T. N. Golemis E. A. (2016). Genomic insights into head and neck cancer. *Cancers Head Neck* 1:1. 10.1186/s41199-016-0003-z 29034103 PMC5638139

[B7] Bertrand G. Maalouf M. Boivin A. Battiston-Montagne P. Beuve M. Levy A. (2014). Targeting head and neck cancer stem cells to overcome resistance to photon and carbon ion radiation. *Stem Cell Rev. Rep.* 10 114–126. 10.1007/s12015-013-9467-y 23955575

[B8] Billin A. N. Thirlwell H. Ayer D. E. (2000). Beta-catenin-histone deacetylase interactions regulate the transition of LEF1 from a transcriptional repressor to an activator. *Mol. Cell Biol.* 20 6882–6890. 10.1128/mcb.20.18.6882-6890.2000 10958684 PMC88764

[B9] Bordonaro M. Lazarova D. L. (2015). CREB-binding protein, p300, butyrate, and Wnt signaling in colorectal cancer. *World J. Gastroenterol.* 21 8238–8248. 10.3748/wjg.v21.i27.8238 26217075 PMC4507093

[B10] Bordonaro M. Shirasawa S. Lazarova D. L. (2016). In hyperthermia increased ERK and WNT signaling suppress colorectal cancer cell growth. *Cancers* 8:49. 10.3390/cancers8050049 27187477 PMC4880866

[B11] Cancer Genome Atlas Network (2015). Comprehensive genomic characterization of head and neck squamous cell carcinomas. *Nature* 517 576–582. 10.1038/nature14129 25631445 PMC4311405

[B12] Chen D. Wang C. Y. (2019). Targeting cancer stem cells in squamous cell carcinoma. *Precis. Clin. Med.* 2 152–165. 10.1093/pcmedi/pbz016 31598386 PMC6770277

[B13] Cheng A. S. Lau S. S. Chen Y. Kondo Y. Li M. S. Feng H. (2011). EZH2-mediated concordant repression of Wnt antagonists promotes β-catenin-dependent hepatocarcinogenesis. *Cancer Res.* 71 4028–4039. 10.1158/0008-5472.CAN-10-3342 21512140

[B14] Cheng Y. Phoon Y. P. Jin X. Chong S. Y. Ip J. C. Wong B. W. (2015). Wnt-C59 arrests stemness and suppresses growth of nasopharyngeal carcinoma in mice by inhibiting the Wnt pathway in the tumor microenvironment. *Oncotarget* 6 14428–14439. 10.18632/oncotarget.3982 25980501 PMC4546477

[B15] Dawson K. Aflaki M. Nattel S. (2013). Role of the Wnt-Frizzled system in cardiac pathophysiology: a rapidly developing, poorly understood area with enormous potential. *J. Physiol.* 591 1409–1432. 10.1113/jphysiol.2012.235382 23207593 PMC3607163

[B16] De A. (2011). Wnt/Ca2+ signaling pathway: a brief overview. *Acta Biochim. Biophys. Sin.* 43 745–756. 10.1093/abbs/gmr079 21903638

[B17] Emami K. H. Nguyen C. Ma H. Kim D. H. Jeong K. W. Eguchi M. (2004). A small molecule inhibitor of beta-catenin/CREB-binding protein transcription [corrected]. *Proc. Natl. Acad. Sci. U.S.A.* 101 12682–12687. 10.1073/pnas.0404875101 15314234 PMC515116

[B18] Fan J. Wei Q. Liao J. Zou Y. Song D. Xiong D. (2017). Noncanonical Wnt signaling plays an important role in modulating canonical Wnt-regulated stemness, proliferation and terminal differentiation of hepatic progenitors. *Oncotarget* 8 27105–27119. 10.18632/oncotarget.15637 28404920 PMC5432321

[B19] Farooqi A. A. Shu C. W. Huang H. W. Wang H. R. Chang Y. T. Fayyaz S. (2017). TRAIL, Wnt, sonic hedgehog, TGFβ, and miRNA signalings are potential targets for oral cancer therapy. *Int. J. Mol. Sci.* 18:1523. 10.3390/ijms18071523 28708091 PMC5536013

[B20] Filippone M. G. Di Palma T. Lucci V. Zannini M. (2014). Pax8 modulates the expression of Wnt4 that is necessary for the maintenance of the epithelial phenotype of thyroid cells. *BMC Mol. Biol.* 15:21. 10.1186/1471-2199-15-21 25270402 PMC4200477

[B21] Flores-Hernández E. Velázquez D. M. Castañeda-Patlán M. C. Fuentes-García G. Fonseca-Camarillo G. Yamamoto-Furusho J. K. (2020). Canonical and non-canonical Wnt signaling are simultaneously activated by Wnts in colon cancer cells. *Cell. Signal* 72:109636. 10.1016/j.cellsig.2020.109636 32283254

[B22] Foulquier S. Daskalopoulos E. P. Lluri G. Hermans K. C. M. Deb A. Blankesteijn W. M. (2018). WNT signaling in cardiac and vascular disease. *Pharmacol. Rev.* 70 68–141. 10.1124/pr.117.013896 29247129 PMC6040091

[B23] González-Moles M. A. Ruiz-Ávila I. Gil-Montoya J. A. Plaza-Campillo J. Scully C. (2014). β-catenin in oral cancer: an update on current knowledge. *Oral Oncol.* 50 818–824. 10.1016/j.oraloncology.2014.06.005 24998198

[B24] Gordon M. D. Nusse R. (2006). Wnt signaling: multiple pathways, multiple receptors, and multiple transcription factors. *J. Biol. Chem.* 281 22429–22433. 10.1074/jbc.R600015200 16793760

[B25] Hagen T. Vidal-Puig A. (2002). Characterisation of the phosphorylation of beta-catenin at the GSK-3 priming site Ser45. *Biochem. Biophys. Res. Commun.* 294 324–328. 10.1016/S0006-291X(02)00485-0 12051714

[B26] Hayes J. Peruzzi P. P. Lawler S. (2014). MicroRNAs in cancer: biomarkers, functions and therapy. *Trends Mol. Med.* 20 460–469. 10.1016/j.molmed.2014.06.005 25027972

[B27] Hernández-Maqueda J. G. Luna-Ulloa L. B. Santoyo-Ramos P. Castañeda-Patlán M. C. Robles-Flores M. (2013). Protein kinase C delta negatively modulates canonical Wnt pathway and cell proliferation in colon tumor cell lines. *PLoS One* 8:e58540. 10.1371/journal.pone.0058540 23520519 PMC3592802

[B28] Hu T. Li C. (2010). Convergence between Wnt-β-catenin and EGFR signaling in cancer. *Mol. Cancer* 9:236. 10.1186/1476-4598-9-236 20828404 PMC2944186

[B29] Huang S. M. Mishina Y. M. Liu S. Cheung A. Stegmeier F. Michaud G. A. (2009). Tankyrase inhibition stabilizes axin and antagonizes Wnt signalling. *Nature* 461 614–620. 10.1038/nature08356 19759537

[B30] Humphries A. C. Mlodzik M. (2018). From instruction to output: Wnt/PCP signaling in development and cancer. *Curr. Opin. Cell Biol.* 51 110–116. 10.1016/j.ceb.2017.12.005 29289896 PMC5949250

[B31] Ishida K. Ito S. Wada N. Deguchi H. Hata T. Hosoda M. (2007). Nuclear localization of beta-catenin involved in precancerous change in oral leukoplakia. *Mol. Cancer* 6:62. 10.1186/1476-4598-6-62 17922924 PMC2140063

[B32] Iwai S. Katagiri W. Kong C. Amekawa S. Nakazawa M. Yura Y. (2005). Mutations of the APC, beta-catenin, and axin 1 genes and cytoplasmic accumulation of beta-catenin in oral squamous cell carcinoma. *J. Cancer Res. Clin. Oncol.* 131 773–782. 10.1007/s00432-005-0027-y 16163548 PMC12161198

[B33] Javed Z. Muhammad Farooq H. Ullah M. Zaheer Iqbal M. Raza Q. Sadia H. (2019). Wnt signaling: a potential therapeutic target in head and neck squamous cell carcinoma. *Asian Pac. J. Cancer Prev.* 20 995–1003. 10.31557/APJCP.2019.20.4.995 31030466 PMC6948882

[B34] Ji P. Zhou Y. Yang Y. Wu J. Zhou H. Quan W. (2019). Myeloid cell-derived LL-37 promotes lung cancer growth by activating Wnt/β-catenin signaling. *Theranostics* 9 2209–2223. 10.7150/thno.30726 31149039 PMC6531301

[B35] Kartha V. K. Alamoud K. A. Sadykov K. Nguyen B. C. Laroche F. Feng H. (2018). Functional and genomic analyses reveal therapeutic potential of targeting β-catenin/CBP activity in head and neck cancer. *Genome Med.* 10:54. 10.1186/s13073-018-0569-7 30029671 PMC6053793

[B36] Katase N. Gunduz M. Beder L. B. Gunduz E. Al Sheikh Ali M. Tamamura R. (2010). Frequent allelic loss of Dkk-1 locus (10q11.2) is related with low distant metastasis and better prognosis in head and neck squamous cell carcinomas. *Cancer Invest.* 28 103–110. 10.3109/07357900903095680 19995224

[B37] Katase N. Lefeuvre M. Gunduz M. Gunduz E. Beder L. B. Grenman R. (2012). Absence of Dickkopf (Dkk)-3 protein expression is correlated with longer disease-free survival and lower incidence of metastasis in head and neck squamous cell carcinoma. *Oncol. Lett.* 3 273–280. 10.3892/ol.2011.473 22740894 PMC3362421

[B38] Katase N. Lefeuvre M. Tsujigiwa H. Fujii M. Ito S. Tamamura R. (2013). Knockdown of Dkk-3 decreases cancer cell migration and invasion independently of the Wnt pathways in oral squamous cell carcinoma-derived cells. *Oncol. Rep.* 29 1349–1355. 10.3892/or.2013.2251 23354949

[B39] Katase N. Nagano K. Fujita S. (2020). DKK3 expression and function in head and neck squamous cell carcinoma and other cancers. *J. Oral Biosci.* 62 9–15. 10.1016/j.job.2020.01.008 32032750

[B40] Kawakita A. Yanamoto S. Yamada S. Naruse T. Takahashi H. Kawasaki G. (2014). MicroRNA-21 promotes oral cancer invasion via the Wnt/β-catenin pathway by targeting DKK2. *Pathol. Oncol. Res.* 20 253–261. 10.1007/s12253-013-9689-y 23999978

[B41] Kestler H. A. Kühl M. (2008). From individual Wnt pathways towards a Wnt signalling network. *Philos. Trans. R. Soc. Lond. B Biol. Sci.* 363 1333–1347. 10.1098/rstb.2007.2251 18192173 PMC2610122

[B42] Kobayashi K. Hisamatsu K. Suzui N. Hara A. Tomita H. Miyazaki T. (2018). A review of HPV-related head and neck cancer. *J. Clin. Med.* 7:241. 10.3390/jcm7090241 30150513 PMC6162868

[B43] Kremenevskaja N. von Wasielewski R. Rao A. S. Schöfl C. Andersson T. Brabant G. (2005). Wnt-5a has tumor suppressor activity in thyroid carcinoma. *Oncogene* 24 2144–2154. 10.1038/sj.onc.1208370 15735754

[B44] Kulak O. Chen H. Holohan B. Wu X. He H. Borek D. (2015). Disruption of Wnt/β-Catenin signaling and telomeric shortening are inextricable consequences of tankyrase inhibition in human cells. *Mol. Cell. Biol.* 35 2425–2435. 10.1128/MCB.00392-315 25939383 PMC4475917

[B45] Lamb R. Ablett M. P. Spence K. Landberg G. Sims A. H. Clarke R. B. (2013). Wnt pathway activity in breast cancer sub-types and stem-like cells. *PLoS One* 8:e67811. 10.1371/journal.pone.0067811 23861811 PMC3701602

[B46] Lammi L. Arte S. Somer M. Jarvinen H. Lahermo P. Thesleff I. (2004). Mutations in AXIN2 cause familial tooth agenesis and predispose to colorectal cancer. *Am. J. Hum. Genet.* 74 1043–1050. 10.1086/386293 15042511 PMC1181967

[B47] Lau T. Chan E. Callow M. Waaler J. Boggs J. Blake R. A. (2013). A novel tankyrase small-molecule inhibitor suppresses APC mutation-driven colorectal tumor growth. *Cancer Res.* 73 3132–3144. 10.1158/0008-5472.CAN-12-4562 23539443

[B48] Le P. N. Keysar S. B. Miller B. Eagles J. R. Chimed T. S. Reisinger J. (2019). Wnt signaling dynamics in head and neck squamous cell cancer tumor-stroma interactions. *Mol. Carcinog.* 58 398–410. 10.1002/mc.22937 30378175 PMC6460915

[B49] Lee C. H. Hung H. W. Hung P. H. Shieh Y. S. (2010). Epidermal growth factor receptor regulates beta-catenin location, stability, and transcriptional activity in oral cancer. *Mol. Cancer* 9:64. 10.1186/1476-4598-9-64 20302655 PMC2850885

[B50] Lee S. H. Koo B. S. Kim J. M. Huang S. Rho Y. S. Bae W. J. (2014). Wnt/β-catenin signalling maintains self-renewal and tumourigenicity of head and neck squamous cell carcinoma stem-like cells by activating Oct4. *J. Pathol.* 4 99–107. 10.1002/path.4383 24871033

[B51] Leethanakul C. Patel V. Gillespie J. Pallente M. Ensley J. F. Koontongkaew S. (2000). Distinct pattern of expression of differentiation and growth-related genes in squamous cell carcinomas of the head and neck revealed by the use of laser capture microdissection and cDNA arrays. *Oncogene* 19 3220–3224. 10.1038/sj.onc.1203703 10918578

[B52] Li N. Li D. Du Y. Su C. Yang C. Lin C. (2019). Overexpressed PLAGL2 transcriptionally activates Wnt6 and promotes cancer development in colorectal cancer. *Oncol. Rep.* 41 875–884. 10.3892/or.2018.6914 30535429 PMC6313070

[B53] Lien W. H. Fuchs E. (2014). Wnt some lose some: transcriptional governance of stem cells by Wnt/β-catenin signaling. *Genes Dev.* 28 1517–1532. 10.1101/gad.244772.114 25030692 PMC4102759

[B54] Lim Y. C. Kang H. J. Kim Y. S. Choi E. C. (2012). All-trans-retinoic acid inhibits growth of head and neck cancer stem cells by suppression of Wnt/β-catenin pathway. *Eur. J. Cancer* 48 3310–3318. 10.1016/j.ejca.2012.04.013 22640830

[B55] Liu H. Li J. Zhou Y. Hu Q. Zeng Y. Mohammadreza M. M. (2017). Human papillomavirus as a favorable prognostic factor in a subset of head and neck squamous cell carcinomas: a meta-analysis. *J. Med. Virol.* 89 710–725. 10.1002/jmv.24670 27575972

[B56] Logan C. Y. Nusse R. (2004). The Wnt signaling pathway in development and disease. *Annu. Rev. Cell Dev. Biol.* 20 781–810. 10.1146/annurev.cellbio.20.010403.113126 15473860

[B57] Lustig B. Behrens J. (2003). The Wnt signaling pathway and its role in tumor development. *J. Cancer Res. Clin. Oncol.* 129 199–221. 10.1007/s00432-003-0431-0 12707770 PMC12161963

[B58] Ma X. Yan W. Dai Z. Gao X. Ma Y. Xu Q. (2016). Baicalein suppresses metastasis of breast cancer cells by inhibiting EMT via downregulation of SATB1 and Wnt/β-catenin pathway. *Drug Des. Devel. Ther.* 10 1419–1441. 10.2147/DDDT.S102541 27143851 PMC4841441

[B59] MacDonald B. T. Tamai K. He X. (2009). Wnt/beta-catenin signaling: components, mechanisms, and diseases. *Dev. Cell* 17 9–26. 10.1016/j.devcel.2009.06.016 19619488 PMC2861485

[B60] Mannelli G. Gallo O. (2012). Cancer stem cells hypothesis and stem cells in head and neck cancers. *Cancer Treat Rev.* 38 515–539. 10.1016/j.ctrv.2011.11.007 22197808

[B61] Morin P. J. Sparks A. B. Korinek V. Barker N. Clevers H. Vogelstein B. (1997). Activation of beta-catenin-Tcf signaling in colon cancer by mutations in beta-catenin or APC. *Science* 275 1787–1790. 10.1126/science.275.5307.1787 9065402

[B62] Ng L. F. Kaur P. Bunnag N. Suresh J. Sung I. C. H. Tan Q. H. (2019). WNT Signaling in Disease. *Cells* 8:826. 10.3390/cells8080826 31382613 PMC6721652

[B63] Ng O. H. Erbilgin Y. Firtina S. Celkan T. Karakas Z. Aydogan G. (2014). Deregulated WNT signaling in childhood T-cell acute lymphoblastic leukemia. *Blood Cancer J.* 4:e192. 10.1038/bcj.2014.12 24632884 PMC3972698

[B64] Nusse R. Brown A. Papkoff J. Scambler P. Shackleford G. McMahon A. (1991). A new nomenclature for int-1 and related genes: the Wnt gene family. *Cell* 64:231 10.1016/0092-8674(91)90633-a 1846319

[B65] Nwanze J. Cohen C. Schmitt A. C. Siddiqui M. T. (2015). β-Catenin expression in oropharyngeal squamous cell carcinomas: comparison and correlation with p16 and human papillomavirus in situ hybridization. *Acta Cytol.* 59 479–484. 10.1159/000443602 26849661

[B66] Paluszczak J. Sarbak J. Kostrzewska-Poczekaj M. Kiwerska K. Jarmuż-Szymczak M. Grenman R. (2015). The negative regulators of Wnt pathway-DACH1, DKK1, and WIF1 are methylated in oral and oropharyngeal cancer and WIF1 methylation predicts shorter survival. *Tumour Biol.* 36 2855–2861. 10.1007/s13277-014-2913-x 25487617 PMC4428535

[B67] Pannone G. Bufo P. Santoro A. Franco R. Aquino G. Longo F. (2010). WNT pathway in oral cancer: epigenetic inactivation of WNT-inhibitors. *Oncol. Rep.* 24 1035–1041. 10.3892/or.2010.1035 20811686

[B68] Peng L. Li Y. Shusterman K. Kuehl M. Gibson C. W. (2011). Wnt-RhoA signaling is involved in dental enamel development. *Eur. J. Oral Sci.* 119(Suppl. 1), 41–49. 10.1111/j.1600-0722.2011.00880.x 22243225 PMC3270888

[B69] Polakis P. (2012). Wnt signaling in cancer. *Cold Spring Harb. Perspect. Biol.* 4:a008052. 10.1101/cshperspect.a008052 22438566 PMC3331705

[B70] Prgomet Z. Andersson T. Lindberg P. (2017). Higher expression of WNT5A protein in oral squamous cell carcinoma compared with dysplasia and oral mucosa with a normal appearance. *Eur. J. Oral Sci.* 125 237–246. 10.1111/eos.12352 28603941 PMC5519933

[B71] Prgomet Z. Axelsson L. Lindberg P. Andersson T. (2015). Migration and invasion of oral squamous carcinoma cells is promoted by WNT5A, a regulator of cancer progression. *J. Oral Pathol. Med.* 44 776–784. 10.1111/jop.12292 25459554

[B72] Prince M. E. Sivanandan R. Kaczorowski A. Wolf G. T. Kaplan M. J. Dalerba P. (2007). Identification of a subpopulation of cells with cancer stem cell properties in head and neck squamous cell carcinoma. *Proc. Natl. Acad. Sci. U.S.A.* 104 973–978. 10.1073/pnas.0610117104 17210912 PMC1783424

[B73] Proffitt K. D. Madan B. Ke Z. Pendharkar V. Ding L. Lee M. A. (2013). Pharmacological inhibition of the Wnt acyltransferase PORCN prevents growth of WNT-driven mammary cancer. *Cancer Res.* 73 502–507. 10.1158/0008-5472.CAN-12-2258 23188502

[B74] Psyrri A. Kotoula V. Fountzilas E. Alexopoulou Z. Bobos M. Televantou D. (2014). Prognostic significance of the Wnt pathway in squamous cell laryngeal cancer. *Oral Oncol.* 50 298–305. 10.1016/j.oraloncology.2014.01.005 24461629

[B75] Qin L. Yin Y. T. Zheng F. J. Peng L. X. Yang C. F. Bao Y. N. (2015). WNT5A promotes stemness characteristics in nasopharyngeal carcinoma cells leading to metastasis and tumorigenesis. *Oncotarget* 6 10239–10252. 10.18632/oncotarget.3518 25823923 PMC4496352

[B76] Rao T. P. Kühl M. (2010). An updated overview on Wnt signaling pathways: a prelude for more. *Circ. Res.* 106 1798–1806. 10.1161/CIRCRESAHA.110.219840 20576942

[B77] Rhee C. S. Sen M. Lu D. Wu C. Leoni L. Rubin J. (2002). Wnt and frizzled receptors as potential targets for immunotherapy in head and neck squamous cell carcinomas. *Oncogene* 21 6598–6605. 10.1038/sj.onc.1205920 12242657

[B78] Rubinfeld B. Robbins P. El-Gamil M. Albert I. Porfiri E. Polakis P. (1997). Stabilization of beta-catenin by genetic defects in melanoma cell lines. *Science* 275 1790–1792. 10.1126/science.275.5307.1790 9065403

[B79] Rudy S. F. Brenner J. C. Harris J. L. Liu J. Che J. Scott M. V. (2016). In vivo Wnt pathway inhibition of human squamous cell carcinoma growth and metastasis in the chick chorioallantoic model. *J. Otolaryngol. Head Neck Surg.* 45:26. 10.1186/s40463-016-0140-8 27117272 PMC4845503

[B80] Russell J. O. Monga S. P. (2018). Wnt/β-Catenin signaling in liver development, homeostasis, and pathobiology. *Annu. Rev. Pathol.* 13 351–378. 10.1146/annurev-pathol-020117-044010 29125798 PMC5927358

[B81] Sakamoto T. Kawano S. Matsubara R. Goto Y. Jinno T. Maruse Y. (2017). Critical roles of Wnt5a-Ror2 signaling in aggressiveness of tongue squamous cell carcinoma and production of matrix metalloproteinase-2 via ΔNp63β-mediated epithelial-mesenchymal transition. *Oral Oncol.* 69 15–25. 10.1016/j.oraloncology.2017.03.019 28559016

[B82] Sakisaka Y. Tsuchiya M. Nakamura T. Tamura M. Shimauchi H. Nemoto E. (2015). Wnt5a attenuates Wnt3a-induced alkaline phosphatase expression in dental follicle cells. *Exp. Cell Res.* 336 85–93. 10.1016/j.yexcr.2015.06.013 26112214

[B83] Saneyoshi T. Kume S. Amasaki Y. Mikoshiba K. (2002). The Wnt/calcium pathway activates NF-AT and promotes ventral cell fate in Xenopus embryos. *Nature* 417 295–299. 10.1038/417295a 12015605

[B84] Satoh S. Daigo Y. Furukawa Y. Kato T. Miwa N. Nishiwaki T. (2000). AXIN1 mutations in hepatocellular carcinomas, and growth suppression in cancer cells by virus-mediated transfer of AXIN1. *Nat. Genet.* 24 245–250. 10.1038/73448 10700176

[B85] Shiah S. G. Hsiao J. R. Chang W. M. Chen Y. W. Jin Y. T. Wong T. Y. (2014). Downregulated miR329 and miR410 promote the proliferation and invasion of oral squamous cell carcinoma by targeting Wnt-7b. *Cancer Res.* 74 7560–7572. 10.1158/0008-5472.CAN-14-0978 25351956

[B86] Shiah S. G. Shieh Y. S. Chang J. Y. (2016). The role of Wnt signaling in squamous cell carcinoma. *J. Dent. Res.* 95 129–134. 10.1177/0022034515613507 26516128

[B87] Song J. Chang I. Chen Z. Kang M. Wang C. Y. (2010). Characterization of side populations in HNSCC: highly invasive, chemoresistant and abnormal Wnt signaling. *PLoS One* 5:e11456. 10.1371/journal.pone.0011456 20625515 PMC2897893

[B88] Takeshita A. Iwai S. Morita Y. Niki-Yonekawa A. Hamada M. Yura Y. (2014). Wnt5b promotes the cell motility essential for metastasis of oral squamous cell carcinoma through active Cdc42 and RhoA. *Int. J. Oncol.* 44 59–68. 10.3892/ijo.2013.2172 24220306

[B89] Tejeda-Muñoz N. Robles-Flores M. (2015). Glycogen synthase kinase 3 in Wnt signaling pathway and cancer. *IUBMB Life* 67 914–922. 10.1002/iub.1454 26600003

[B90] Valenta T. Hausmann G. Basler K. (2012). The many faces and functions of β-catenin. *EMBO J.* 31 2714–2736. 10.1038/emboj.2012.150 22617422 PMC3380220

[B91] van Tienen F. H. Laeremans H. van der Kallen C. J. Smeets H. J. (2009). Wnt5b stimulates adipogenesis by activating PPARgamma, and inhibiting the beta-catenin dependent Wnt signaling pathway together with Wnt5a. *Biochem. Biophys. Res. Commun.* 387 207–211. 10.1016/j.bbrc.2009.07.004 19577541

[B92] Velázquez D. M. Castañeda-Patlán M. C. Robles-Flores M. (2017). Dishevelled stability is positively regulated by PKCζ-mediated phosphorylation induced by Wnt agonists. *Cell. Signal* 35 107–117. 10.1016/j.cellsig.2017.03.023 28366812

[B93] Vermeulen L. De Sousa E. Melo F. van der Heijden M. Cameron K. de Jong J. H. (2010). Wnt activity defines colon cancer stem cells and is regulated by the microenvironment. *Nat. Cell Biol.* 12 468–476. 10.1038/ncb2048 20418870

[B94] Vlad A. Röhrs S. Klein-Hitpass L. Müller O. (2008). The first five years of the Wnt targetome. *Cell. Signal* 20 795–802. 10.1016/j.cellsig.2007.10.031 18160255

[B95] Wang C. Zhao Y. Su Y. Li R. Lin Y. Zhou X. (2013). C-Jun N-terminal kinase (JNK) mediates Wnt5a-induced cell motility dependent or independent of RhoA pathway in human dental papilla cells. *PLoS One* 8:e69440. 10.1371/journal.pone.0069440 23844260 PMC3700942

[B96] Warrier S. Bhuvanalakshmi G. Arfuso F. Rajan G. Millward M. Dharmarajan A. (2014). Cancer stem-like cells from head and neck cancers are chemosensitized by the Wnt antagonist, sFRP4, by inducing apoptosis, decreasing stemness, drug resistance and epithelial to mesenchymal transition. *Cancer Gene Ther.* 21 381–388. 10.1038/cgt.2014.42 25104726

[B97] Wong S. H. M. Fang C. M. Chuah L. H. Leong C. O. Ngai S. C. (2018). E-cadherin: its dysregulation in carcinogenesis and clinical implications. *Crit. Rev. Oncol. Hematol.* 121 11–22. 10.1016/j.critrevonc.2017.11.010 29279096

[B98] Wu D. Pan W. (2010). GSK3: a multifaceted kinase in Wnt signaling. *Trends Biochem. Sci.* 35 161–168. 10.1016/j.tibs.2009.10.002 19884009 PMC2834833

[B99] Xi S. Grandis J. R. (2003). Gene therapy for the treatment of oral squamous cell carcinoma. *J. Dent. Res.* 82 11–16. 10.1177/154405910308200104 12508038

[B100] Xie H. Ma Y. Li J. Chen H. Xie Y. Chen M. (2020). WNT7A promotes EGF-induced migration of oral squamous cell carcinoma cells by activating β-catenin/mmp9-mediated signaling. *Front. Pharmacol.* 11:98. 10.3389/fphar.2020.00098 32174831 PMC7054863

[B101] Yao C. J. Lai G. M. Yeh C. T. Lai M. T. Shih P. H. Chao W. J. (2013). Honokiol eliminates human oral cancer stem-like cells accompanied with suppression of Wnt/β -catenin signaling and apoptosis induction. *Evid Based Complement Alternat. Med.* 2013:146136. 10.1155/2013/146136 23662112 PMC3638590

[B102] Yao C. J. Lai G. M. Yeh C. T. Lai M. T. Shih P. H. Chao W. J. (2017). Corrigendum to “Honokiol eliminates human oral cancer stem-like cells accompanied with suppression of Wnt/β-catenin signaling and apoptosis induction”. *Evid Based Complement Alternat. Med.* 2017:9387837. 10.1155/2017/9387837 29358973 PMC5735666

[B103] Yu C. Wang Y. Li G. She L. Zhang D. Chen X. (2018). LncRNA PVT1 promotes malignant progression in squamous cell carcinoma of the head and neck. *J. Cancer.* 9 3593–3602. 10.7150/jca.26465 30310517 PMC6171028

[B104] Zhang C. Hao Y. Sun Y. Liu P. (2019). Quercetin suppresses the tumorigenesis of oral squamous cell carcinoma by regulating microRNA-22/WNT1/β-catenin axis. *J. Pharmacol. Sci.* 140 128–136. 10.1016/j.jphs.2019.03.005 31257059

[B105] Zhang D. Li G. Chen X. Jing Q. Liu C. Lu S. (2019). Wnt3a protein overexpression predicts worse overall survival in laryngeal squamous cell carcinoma. *J. Cancer* 10 4633–4638. 10.7150/jca.35009 31528227 PMC6746142

[B106] Zhang W. Yan Y. Gu M. Wang X. Zhu H. Zhang S. (2017). High expression levels of Wnt5a and Ror2 in laryngeal squamous cell carcinoma are associated with poor prognosis. *Oncol. Lett.* 14 2232–2238. 10.3892/ol.2017.6386 28781662 PMC5530173

[B107] Zheng X. L. Yu H. G. (2018). Wnt6 contributes tumorigenesis and development of colon cancer via its effects on cell proliferation, apoptosis, cell-cycle and migration. *Oncol. Lett.* 6 1163–1172. 10.3892/ol.2018.8729 29963191 PMC6019939

[B108] Zhu H. H. Zhu X. Y. Zhou M. H. Cheng G. Y. Lou W. H. (2014). Effect of WNT5A on epithelial-mesenchymal transition and its correlation with tumor invasion and metastasis in nasopharyngeal carcinoma. *Asian Pac. J. Trop. Med.* 7 488–491. 10.1016/S1995-7645(14)60080-8 25066400

